# Evaluating the accuracy of nine canopy resistance models in estimating winter wheat evapotranspiration using the Penman–Monteith equation

**DOI:** 10.3389/fpls.2024.1470409

**Published:** 2024-11-07

**Authors:** Yingnan Wu, Qiaozhen Li, Xiuli Zhong, Xiaoying Liu

**Affiliations:** Institute of Environment and Sustainable Development in Agriculture, Chinese Academy of Agricultural Sciences, Beijing, China

**Keywords:** Bowen ratio energy balance, winter wheat, evapotranspiration, canopy resistance model, calibration, model parameter, Penman-Monteith equation

## Abstract

Accurate estimation of farmland evapotranspiration (ET) is crucial for agricultural production. The accuracy of the widely used Penman–Monteith (PM) equation for estimating crop ET depends on the quality of input data and their ability to accurately model the canopy resistance (*r*_c_). In this study, we evaluated the PM equation in estimating winter wheat ET using nine *r*_c_ models, with both original and recalibrated parameters, including the Farias (FA), Monteith (MT), Garcίa-Santos (GA), Idso (IS), Jarvis (JA), Katerji-Perrier (KP), Stannard (ST), Todorovic (TD), and Coupled surface resistance (CO) models. We used long-term measurements (2018 to 2023) from the Bowen ratio energy balance method at both daily and seasonal scales. Parameterization was performed using data from the 2020–2021 growing season, while the remaining 4 years were used for verification. The results showed that the FA, KP, and ST models performed better in estimating daily ET with original parameters, achieving a root mean square error (RMSE) of 1.07–1.16 mm d^−1^ and a mean bias error (MBE) of −0.59–0.02 mm d^−1^. After parameterization, the performance of acceptable *r*_c_ models based on RMSE (ranging from 1.07 to 1.22 mm d^−1^, averaged 1.16 mm d^−1^) ranked as follows on the daily scale: FA > CO > KP > ST > IS > GA > JA > MT. The *r*_c_ models were more accurate in simulating ET on a seasonal scale than on the daily scale. Before calibration, the acceptable FA, KP, and MT models overestimated seasonal ET with the MBE ranging from 2.83 to 75.32 mm and RMSE from 29.79 to 82.38 mm. After correction, the suitable *r*_c_ models based on RMSE values decreased by FA > CO > KP > IS > ST > GA > JA on the seasonal scale, which ranged from 29.79 to 76.35 mm. The performance of the revised *r*_c_ models improved on both daily and seasonal scales, with RMSE reductions of 29.03% and 68.18%, respectively. Considering both the accuracy and calculation complexity, the FA and KP models were recommended to be used in the PM equation to estimate daily and seasonal ET in semiarid regions. The CO, GA, ST, IS, and JA models can also be used as alternatives, depending on the availability of meteorological parameters.

## 1 Introduction

Evapotranspiration (ET) is one of the largest components of surface water loss (Zhang et al., 2016), and its accurate determination is essential in areas of water cycle regulation, energy transfer, vegetation health and growth, agricultural water management, hydrological modeling, water resource allocation, ecosystem functioning, and climate change impact assessment (Shen et al., 2002; Sun et al., 2007; Zhang et al., 2022b).

Although ET can be directly measured using several methods, such as weighing lysimeters (Shahrokhnia and Sepaskhah, 2012), eddy covariance (Xu et al., 2021), Bowen ratio energy balance (BREB) (Han and Li, 2010), and sap flow (Zheng et al., 2022), these methods were often limited by high costs, complicated operations, and strict site requirements (Liu et al., 2009). Therefore, accurately estimating ET using meteorological data and empirical or semi-empirical models, which were cost-effective and easier to operate, is crucial. To date, several methods for estimating ET had been well developed, including the one-step approach (López-Urrea and Chávez, 2019), the two-step approach (Meng et al., 2021), and the complimentary relationship approach (Zhou et al., 2015). The Penman–Monteith (PM) equation, recommended by the Food and Agriculture Organization, had reasonable accuracy (Allen et al., 1998). However, its accuracy was heavily dependent on the precise estimation of the canopy resistance (*r*_c_), which varied with crop type, growth stage, and environmental conditions (Irmak et al., 2013). Therefore, a thorough understanding of the canopy resistance was a crucial step when applying the PM equation.

Canopy resistance represented the combined resistance to water vapor through crop leaf stomata, soil resistance to evaporation, and vapor flux resistance under the crop canopy (Lovelli et al., 2008). Parameterizing and directly measuring *r*_c_ was extremely difficult, as it could be influenced by many factors, including solar radiation, air temperature, vapor pressure deficit, soil moisture content, and leaf area (Irmak and Mutiibwa, 2010). Current efforts to parameterize *r*_c_ mainly included the upscaling method (Xu et al., 2018), the inverse method (Niyogi et al., 2008), and the environmental factor function method (Wu et al., 2022).

Various *r*_c_ parameterization models have been proposed. Using hourly BREB measurements data from four typical sunny days, Yan et al. (2020) recalibrated the Katerji-Perrier (KP) parameters and compared the KP and Todorovic (TD) model in the PM, demonstrating satisfactory accuracy. Perez et al. (2005) selected 3 days of 1 year’s data for calibration and Katerji et al. (2011) chose two daytime hourly datasets; they also compared these models on a grass surface and reported that the KP model performed better, while the TD model was not suitable for irrigated grass. Chen et al. (2022) used 1-year data for recalibration and 1-year data for validation and evaluated five *r*_c_ models in estimating maize ET. They found that the Jarvis (JA) model tended to underestimate, with the threshold for over- to underestimation occurring at LAI = 2. When LAI was less than 2, the KP model performed the best. However, when LAI was greater than 2, the KP model underestimated ET. Liu et al. (2011) observed temporal variations in the TD model performance over winter wheat fields. Hourly assessments revealed limited concordance when the field was not fully vegetated, contrasting with strong agreement under full coverage. Li et al. (2015) investigated 11 *r*_c_ models whose parameters were calibrated by 1-year data, to estimate long-term ET for maize and grapevine under sparse and full coverage, indicating that the Coupled surface resistance (CO) model was the most accurate. The calibration of three *r*_c_ models (the KP, TD, and JA) using the PM equation to estimate maize ET showed that the TD and JA models produced reliable results, while the KP model could be used as an alternative (Srivastava et al., 2018).

As described above, knowledge gap remained regarding how they affect the accuracy of the PM estimates and how to select a suitable *r*_c_ model among the numerous models, as evidenced by inconsistent results in literatures. Clearly, the applicability of the *r*_c_ models in the PM equation varies across different regions and under different crops covered. Furthermore, it was common for the same *r*_c_ model that applied the PM equation with the same crop to have different model parameters adopted by different researchers (Xu et al., 2017). For example, Yan et al. (2020) suggested KP model parameters of 0.59 and 0.12 for the winter wheat, while Wang et al. (2016) suggested values of 1.4 and 0.8, respectively. Moreover, most previous studies employed limited datasets, such as a few days or daytime periods, for both parameter calibration and model validation, raising concerns about the model’s applicability, particularly when used across the whole growing season under varied experimental conditions (Spank et al., 2016; Yan et al., 2020; Chen et al., 2022). Previous studies have all directly calculated ET and compared it with the measured ET after calibrating the parameters when using the *r*_c_ models, without calibrating the existing model parameters (Gharsallah et al., 2013; Li et al., 2015; Yan et al., 2020). Verifying whether the previous model parameters can be used may be an indispensable process, because if the previous model parameters are still applicable, parameter calibration may be a redundant process. In order to ensure accuracy in simulating ET when applying *r*_c_ models to the PM equation, long-term data should be used for calibration and verification. At the same time, four criteria were considered in selecting *r*_c_ models: simple form, easily accessible input data, wide applicability, and good performance in previous studies.

The overall objective of this research was to evaluate the performance of nine *r*_c_ models, with both original and calibrated parameters, in applying the PM equation to estimate winter wheat ET at two time scales, i.e., daily and seasonal, using long-term observations from the BREB from a semiarid site. Specifically, the aims were (i) to evaluate the performance of the *r*_c_ models with original parameters to test their universality, and (ii) to examine if parameter calibration could improve the accuracy of the PM equation.

## 2 Materials and methods

### 2.1 Experimental site description

The experiment was conducted at the research base of the Institute of Environment and Sustainable Development in Agriculture, Chinese Academy of Agricultural Sciences, located in Shunyi District, Beijing in northern China (40.09°N, 116.92°E, 33 m a.s.l). The region had a semiarid climate characterized by four distinct seasons. The whole base covered an area of 1,000 ha and was dominated by a homogeneous planting pattern of winter wheat–summer maize rotation. The long-term yearly averaged elements included the following: precipitation of 584 mm, air temperature of 12.6°C, sunshine duration of 7 h, wind speed of 1.60 m·s^−1^, and approximately 200 frost-free days. The soil type was fluvo-aquic, with a field water holding capacity averaging 0.38 cm^3^·cm^−3^ within a soil depth of 1.8 m.

### 2.2 Bowen ratio energy balance method

The BREB was an indirect method for measuring ET, proposed by Bowen in 1926 based on the theory that one-dimensional fluxes of sensible and latent heat could be described in terms of flux–gradient relationships (Bowen, 1926). The one-dimensional surface energy balance equation was as follows:


(1) $$R_{n}=LE+H+G$$

where *R*_n_ is the net radiation flux on the crop surface (W·m^−2^); *LE* is the latent heat flux (W·m^−2^); *H* is the sensible heat flux (W·m^−2^); and *G* is the soil heat flux (W·m^−2^).

Bowen defined the Bowen ratio (*β*) as:


(2) $$\beta =\frac{H}{LE}=\frac{\rho_{a}C_{p}K_{h}\Delta T/\Delta z}{\lambda \rho_{a}\epsilon K_{w}\Delta e/\Delta z}=\gamma \frac{K_{h}\Delta T/\Delta z}{K_{w}\Delta e/\Delta z}$$

where *ρ*_a_ is the air density (kg·m^−3^); *C*_p_ is the air heat capacity; *ϵ* is the ratio of the molecular weight of water to that of dry air (0.622); *K*_w_ and *K*_h_ are the eddy transfer coefficient for latent turbulent and sensible heat (m^2^·s^−1^); Δ*T* and Δ*e* are the difference of potential temperature and water vapor pressure difference between the two measurement altitudes, respectively; Δ*z* is the difference in height; *γ* is the psychrometric constant (kPa·°C^−1^); and *λ* is the latent heat of vaporization (MJ kg^−1^).

By invoking Reynold’s analogy, assuming *K*_w_ = *K*_h_, and steady-state conditions, the Bowen ratio reduced to:


(3) $$\beta =\frac{C_{p}}{\lambda }\frac{\text{Δ}T}{\text{Δ}e}=\gamma \frac{\text{Δ}T}{\text{Δ}e}$$

Combining Equations 1 and 3 results in the following equation to calculate *LE* and *H* by:


(4) $$LE=\left(R_{n}-G\right)/\left(1+\beta \right)$$

(5) $$H=\left(R_{n}-G\right)\beta /\left(1+\beta \right)$$

### 2.3 Bowen ratio system and data collection

The winter wheat field plot covered an area of 0.33 ha, with the BREB system installed near the center of the whole base. The experimental block was surrounded by the same crop (winter wheat and summer maize crop rotation), ensuring that the required fetch was fully satisfied in all directions. The meteorological parameters required in the models were measured by the BREB, and all sensors were installed on a stable tripod. The atmospheric temperature (*T*_a_) and humidity (RH) were measured by two combined polymer capacitive humidity and temperature sensors (HMP 155A-L, Vaisala) mounted at 0.5 m and 2.0 m on two masts extending westward. The canopy temperature (*T*_c_) was measured by the canopy temperature sensor (Apogee, SI-111) mounted on a southward mast at a height of 2 m. The net radiation (*R*_n_) and total radiation (*R*_s_) were measured using the radiation sensor (CNR4, Kipp & Zonen) installed in the same direction and height as the canopy resistance sensors. The wind speed (*u*) was measured by an anemometer (05103L, R.M. Young) installed at the top of the tripod. The sunshine hours, atmospheric pressure (*P*), and rainfall were measured by the sunshine duration sensor (CSD3, Kipp & Zonen), air pressure sensor (PTB110, Vaisala), and tipping bucket rain gauge sensor (TE525MM, Texas Electronics), respectively, erected below the wind speed sensor at a height of 2 m. The soil heat flux and soil temperature were measured by the soil heat flux sensor (HFP01SC, Hukse flux) and soil temperature sensor (109SS, Campbell Scientific) buried 0.1 m underground. The soil water content was measured using soil moisture sensors (CS616, Campbell Scientific) buried at depths of 0.1, 0.25, 0.50, 0.75, and 1.0 m underground, respectively. All meteorological sensors were calibrated at the National Meteorological Center before installation. The data logger (CR1000, Campbell Scientific) was mounted at the midpoint of the tripod. It sampled sensors every 2 s and recorded the 30-min averages, with the system being supervised once a week. The measured data were used to calculate LE (i.e., winter wheat ET) using Equation 4 and recorded as ET-(BREB) for comparison with other methods. The data were filtered by the quality control based on the criteria proposed by Unland et al. (1996), and gaps were filled according to the method outlined by Qiu et al. (2019). Measurements were conducted from 24 March 2018 to 31 July 2023, covering five growing seasons.

### 2.4 Winter wheat characteristics

The winter wheat (*Triticum aestivum* L. of *ZHONGMAI 36*) was seeded around 1 October and harvested around 15 June of the following year, from 2018 to 2023. The winter wheat was mechanically seeded with a row spacing of 15 cm and a planting density of 300 kg·ha^−1^. The cultural practices such as fertilization, weeding, and pesticide were kept uniform throughout the study period, but irrigation varied across the five seasons (see Section 3.1). The physiological indicators used to estimate winter wheat ET included plant height (*h*), leaf area index (*LAI*), and leaf stomatal conductance (*r*_I_), measured every 5–7 days using a ruler, the SS1 Sunscan Canopy Analysis System (Delta-T Devices, England), and the SC-1 Leaf Porometer (Meter, USA), respectively.

**Figure 1:**
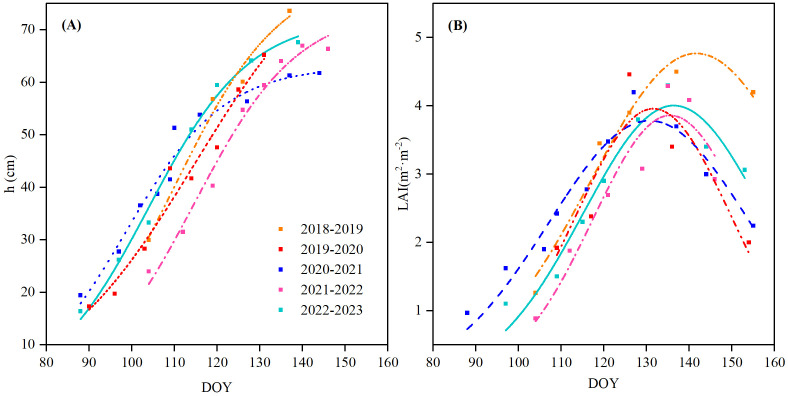
Nonlinear regression of **(A)** plant height (*h*) and **(B)** leaf area index (LAI) of winter wheat from the 2018 to 2023 growth period.

**Figure 2:**
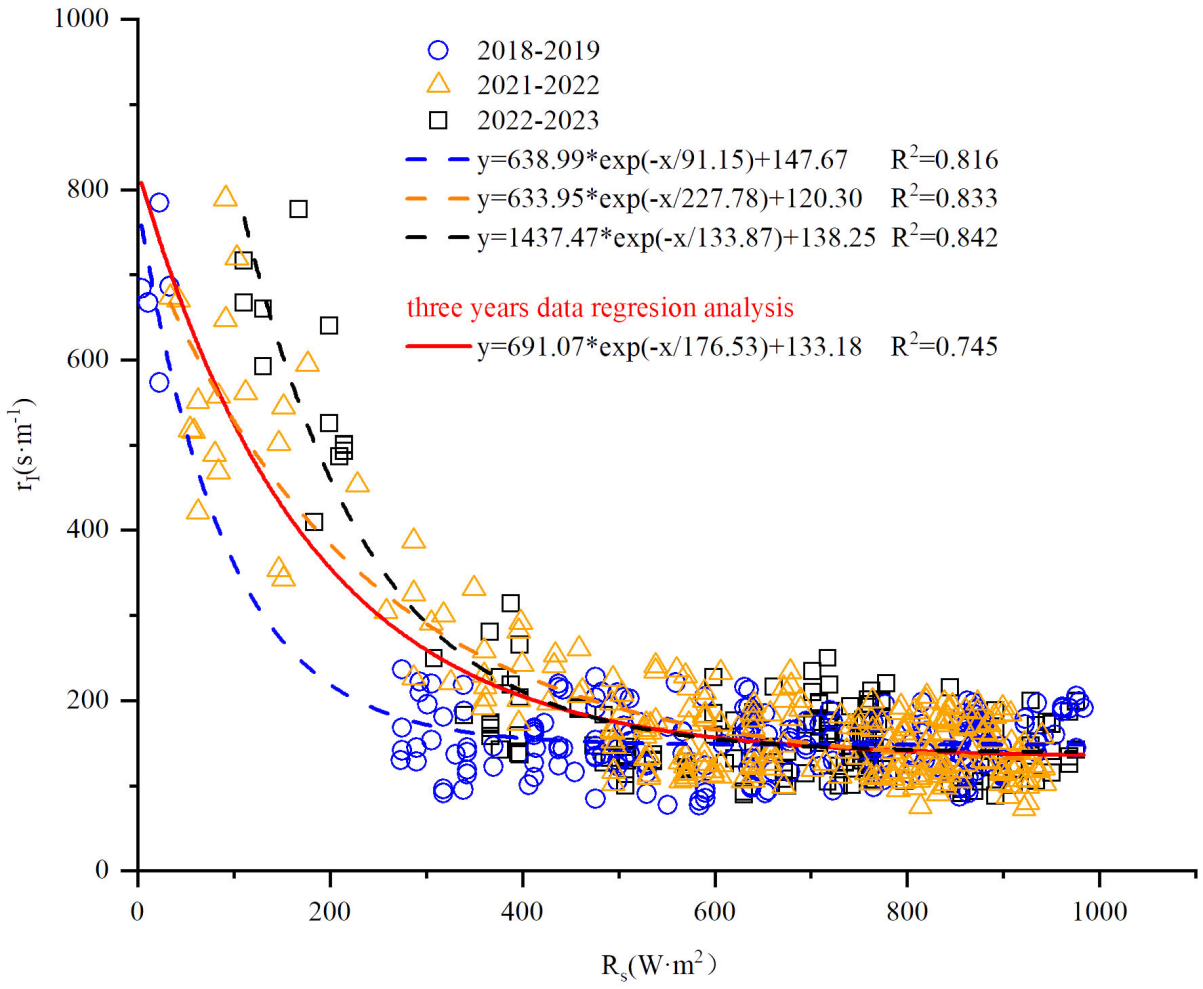
Relationship between leaf stomatal resistance (*r*_I_) and solar radiation (*R*_s_).

Daily data of winter wheat variables (*h*, *LAI*, and *r*_I_) were needed for daily evaluation, but these were measured at longer time intervals as mentioned above. In order to obtain daily values, nonlinear regressions were performed, as shown in **Figures 1** and **2**. Daily *h* was interpolated using a logistic growth model, which demonstrated high accuracy, with all the coefficients of determination (*R* ^2^) exceeding 0.96 (**Figure 1A**). The maximum heights (73.6, 65.2, 61.7, 66.9, and 67.9 cm for each season, respectively) were reached at the flowering stage and remained nearly constant thereafter. Daily LAI followed a downward-opening parabolic curve, with maximum values of 4.5, 4.46, 5.2, 4.2, and 4.3 m^2^ m^−2^ for the five seasons, respectively (**Figure 1B**), occurring approximately at the flowering stage. *r*_I_ showed the strongest regression relationship with *R*_s_ among the examined, including *R*_n_, *T*_a_, and *T*_c_ (**Figure 2**). Note that the relationship used for 2019–2020 and 2020–2021 was the average from the other three seasons due to missing measurements.

### 2.5 Penman–Monteith equation

The estimation of winter wheat ET was based on the PM equation (Penman, 1948). It could be described as follows:


(6) $$\lambda ET=\frac{\Delta \left(R_{n}-G\right)+\rho_{a}C_{p}VPD/r_{a}}{\Delta +\gamma \left(1+r_{c}/r_{a}\right)}$$

where *λET* is the crop ET (W m^−2^); Δ is the slope of the curve when the saturated water vapor pressure is at air temperature (kPa °C^−1^); *γ* is the psychrometric constant (kPa °C^−1^); *R*_n_ is the net radiation flux on the crop surface (W m^−2^); *G* is the soil surface heat flux (W m^−2^); *ρ*_a_ is the air density (kg m^−3^); *C*_p_ is the specific heat capacity of air constant pressure (J kg^−1^ °C^−1^); *VPD* is the water vapor pressure deficit (kPa); *r*_a_ is the aerodynamic resistance (s m^−1^); and *r*_c_ is the crop canopy resistance (s m^−1^), calculated by *r*_c_ models.

The aerodynamic resistance (*r*_a_) can be described as Perrier (1975):


(7) $$r_{a}=\frac{\ln[\left(z-d\right)/z_{0}]\ln[\left(z-d\right)/\left(h-d\right)]}{k^{2}u_{z}}$$

where *u_z_* is wind speed of *z* meters (m s^−1^); *z* is measuring height (*z* = 2 m); *k* is the Von Karman constant with a value of 0.41; and *d*, *z*_0_, and *h* are zero plane displacement, roughness length of controlling momentum transfer, and crop canopy height (m), respectively. Following Allen et al. (1998), their relationship was:


(8) $$d=2/3\;h;z_{0}=0.123\;h$$

### 2.6 Canopy resistance models

In this paper, nine *r*_c_ models were applied to the PM equation to simulate ET of winter wheat. These models included the upscaling method [Monteith (MT), Idso (IS), Farias (FA), KP, and TD] and the environmental factor function method [CO model, JA, Stannard (ST), and Garcίa-Santos (GA)]. Six of the *r*_c_ models (expect FA, MT, and TD) required the parameter recalibration, which used only daytime (9:00–15:00) data (*n* = 3,549) measured by the BREB. All parameters were optimized using the least squares method through the MATLAB. The 30-min all-day data from four seasons were used to calculate *r*_c_, which was then brought into the PM equation (Equation 6) to estimate wheat ET, denoted as the PM-*r*_c_ model, for example, PM-MT and PM-CO. These estimates were compared with the BREB measurements. Both the original and calibrated parameters of the *r*_c_ models are provided in **Table 1**.

**Table 1:** The list of canopy resistance models, original parameters, and calibrated parameters.

Model	Formula	Original parameter	Resource	Corrected parameter
CO	$\begin{array}{l} r_{c}^{CO}={\left[\left(a_{0}LAI+a_{1}\right)/r_{c}^{JA}+a_{2}/r_{s}\right]}^{-1}\\ r_{s}={\left\{a_{3}+\exp[a_{4}+a_{5}f\left(\theta \right)]\right\}}^{-1}\\ f\left(\theta \right)=\left(\theta_{i}-\theta_{W}\right){\left(\theta_{F}-\theta_{W}\right)}^{-1} \end{array}$	*a*_0_ = −0.15, *a*_1_ = 0.14 a_2_ = 0.01, *a*_3_ = 1.57 *a*_4_ = −2.14, *a*_5_ = −8.96	Li et al. (2014)	*a*_0_ = 0.32, *a*_1_ = −0.75 *a*_2_ = −1.76, *a*_3_ = −0.003 *a*_4_ = −1,192.4, *a*_5_ = 5.27
FA	$\begin{array}{l} r_{c}^{FA}=r_{i}f{\left(\theta \right)}^{-1}\\ r_{i}=\rho_{a}c_{p}VPD{\left[\Delta \left(R_{n}-G\right)\right]}^{-1}\\ f\left(\theta \right)=\left(\theta_{i}-\theta_{W}\right){\left(\theta_{F}-\theta_{W}\right)}^{-1} \end{array}$	–	Ortega-Farias et al. (2004)	–
MT	$\begin{array}{l} r_{c}^{FAO}=r_{I}LA{I_{active}}^{-1}\\ LAI_{active}=\{\begin{array}{ccc} LAI & \;LAI\leq 2 & \\ 2 & 2<LAI<4 & \\ LAI/2 & \;LAI\geq 4 & \end{array} \end{array}$	$r_{c}^{FAO}$ =70	Monteith (1965)	Calibrated by **Figure 3**
GA	$r_{c}^{GA}={\left[b_{1}\frac{\left(1100+b_{2}\right)R_{n}}{1100\left(R_{n}+b_{2}\right)}\exp\left(-b_{3}VPD\right)\right]}^{-1}$	*b*_1_ = 11.8 *b*_2_ = 433.1 *b*_3_ = 0.084	García-Santos et al. (2009)	*b*_1_ = 9.34 *b*_2_ = 325.42 *b*_3_ = 0.35
IS	$\begin{array}{l} r_{c}^{IS}=\left[1-c_{2}\left(\Delta +\gamma \right)\right]{\left(1-c_{2}\Delta \right)}^{-1}\left(c_{1}\rho C_{p}\right){\left(c_{2}\gamma R_{n}\right)}^{-1}\\ T_{c}-T_{a}=c_{1}-c_{2}VPD \end{array}$	*c*_1_ = 1.08 *c*_2_ = 2.09	Idso (1983)	*c*_1_ = 1.53 *c*_2_ = 0.62
JA	$\begin{array}{l} r_{c}^{JA}=r_{c\min}{\left[LAIf\left(R_{n}\right)f\left(VPD\right)f\left(T_{a}\right)f\left(\theta \right)\right]}^{-1}\\ f\left(R_{n}\right)=1-\exp\left(-R_{n}d_{1}^{-1}\right)\\ f\left(T_{a}\right)=1-d_{2}(25-T_{a})2\\ f\left(VPD\right)=1-d_{3}VPD\\ f\left(\theta \right)=\left(\theta_{i}-\theta_{W}\right){\left(\theta_{F}-\theta_{W}\right)}^{-1} \end{array}$	*d*_1_ = 161.8 *d*_2_ = 0.013 *d*_3_ = 0.001	Zhao et al. (2015)	*d*_1_ = 618.96 *d*_2_ = 0.0013 *d*_3_ = 0.001
KP	$\begin{array}{l} r_{c}^{KP}=\left(e_{1}r^{*}r_{a}^{-1}+e_{2}\right)r_{a}\\ r^{*}=\left(\Delta +\gamma \right){\left(\Delta \gamma \right)}^{-1}\rho C_{p}\left(e_{s}-e_{a}\right){\left(R_{n}-G\right)}^{-1} \end{array}$	*e*_1_ = 0.54 *e*_2_ = 0.61	Yan et al. (2020)	*e*_1_ = 0.85 *e*_2_ = −0.29
ST	$\begin{array}{l} r_{c}^{ST}={\left[f(LAI)f(VPD)f(Rn)\right]}^{-1}\\ f(LAI)=f_{1}LAI{\left(LAI_{\max}\right)}^{-1}\\ f(VPD)=f_{2}{\left(f_{2}+VPD\right)}^{-1}\\ f(R_{n})=R_{n}(R_{n\max}+f_{3}){\left[R_{n\max}(R_{n}+f_{3})\right]}^{-1} \end{array}$	*f*_1_ = 0.711 *f*_2_ = 0.00804 *f*_3_ = 41	Stannard (1993)	*f*_1_ = 0.77 *f*_2_ = 0.089 *f*_3_ = 1171
TD	$\begin{array}{l} r_{c}^{TD}=Xr_{i}\\ aX^{2}+bX+c=0\\ a=\left(\Delta +\gamma r_{i}r_{a}^{-1}\right){\left(\Delta +\gamma \right)}^{-1}r_{i}r_{a}^{-1}VPD\\ b=-\gamma r_{i}r_{a}^{-1}\gamma VPD{\left[\Delta \left(\Delta +\gamma \right)\right]}^{-1}\\ c=-\left(\Delta +\gamma \right)\gamma VPD{\left[\Delta \left(\Delta +\gamma \right)\right]}^{-1}\\ r_{i}=\rho_{a}c_{p}VPD{\left[\Delta \left(R_{n}-G\right)\right]}^{-1} \end{array}$	–	Todorovic (1999)	–

*CO*, *FA*, *MT*, *GA*, *IS*, *JA*, *KP*, *ST*, and *TD* represented the Coupled surface, Farias, Monteith, Garcίa-Santos, Idso, Jarvis, Katerji-Perrier, Stannard, and Todorovic canopy resistance models, respectively. *r*_c_ with different superscripts represented the canopy resistance of different models. *a–f* with numerical subscripts represented the parameters in the canopy resistance models, and the numerical subscripts represent the number of parameters required for the canopy resistance models. *r*_s_, *r*_i_, *r*_I,_ and r^*^ represented soil resistance, modified climatological resistance, leaf resistance, and climatic resistance (s·m^−1^), respectively. *θ*_i_, *θ*_W_, and *θ*_F_ represented soil moisture content, wilting coefficient, and field capacity (cm^3^·cm^−3^), respectively. *LAI*_active_ and *LAI*_max_ represented effective leaf area index and maximum leaf area index (m^2^·m^−2^), respectively. *T*_c_ represented canopy temperature (°C). *R*_nmax_ represented maximum net radiation (W·m^−2^). *X* represented the ratio of canopy resistance to climatological resistance. – meant the *r*_c_ model did not need to calibrate. The other symbols were consistent with those shown above.

The uncalibrated *r*_c_ models (i.e., using original parameters) were first evaluated using data from four seasons (expect 2020–2021), aiming to test their universality. Then, models were then calibrated using data from 2020 to 2021 to examine whether parameter calibration could improve the PM’s estimation accuracy of winter wheat ET. In the second-round comparisons with calibrated parameters, the same four-season dataset was used.

### 2.7 Evaluation of model performance

The performance of the *r*_c_ models was evaluated using statistical indicators including root mean square error (RMSE), mean bias error (MBE), the determination coefficient (*R* ^2^), and index of agreement (*d*). They were calculated as:


(9) $$MSE=\sqrt{\frac{1}{n}{\sum_{i=1}^{n}\left(E_{i}-O_{i}\right)}^{2}}$$

(10) $$MBE=\frac{1}{n}\sum_{i=1}^{n}\left(E_{i}-O_{i}\right)$$

(11) $$R^{2}=\frac{{\left(\sum_{i=1}^{n}\left(O_{i}-\bar{O}\right)\left(E_{i}-\bar{E}\right)\right)}^{2}}{\sum_{i=1}^{n}{\left(O_{i}-\bar{O}\right)}^{2}\sum_{i=1}^{n}{\left(E_{i}-\bar{E}\right)}^{2}}$$

(12) $$d=1-\frac{\sum_{i=1}^{n}{\left(E_{i}-O_{i}\right)}^{2}}{\sum_{i=1}^{n}{\left(\left|E_{i}-\bar{O}\right|+\left|O_{i}-\bar{O}\right|\right)}^{2}}$$

where *O_i_* and *E_i_* are the observed and estimated values, respectively; $\bar{O}$ and $\bar{E}$ are their respective average; the subscript *i* is the *i*th value; and *n* is total record of data. Lower values of MBE and RMSE indicate better model performance, and vice versa for the *R* ^2^ and *d*.

## 3 Results and discussion

### 3.1 Meteorological and water condition during experiment

**Figure 3:**
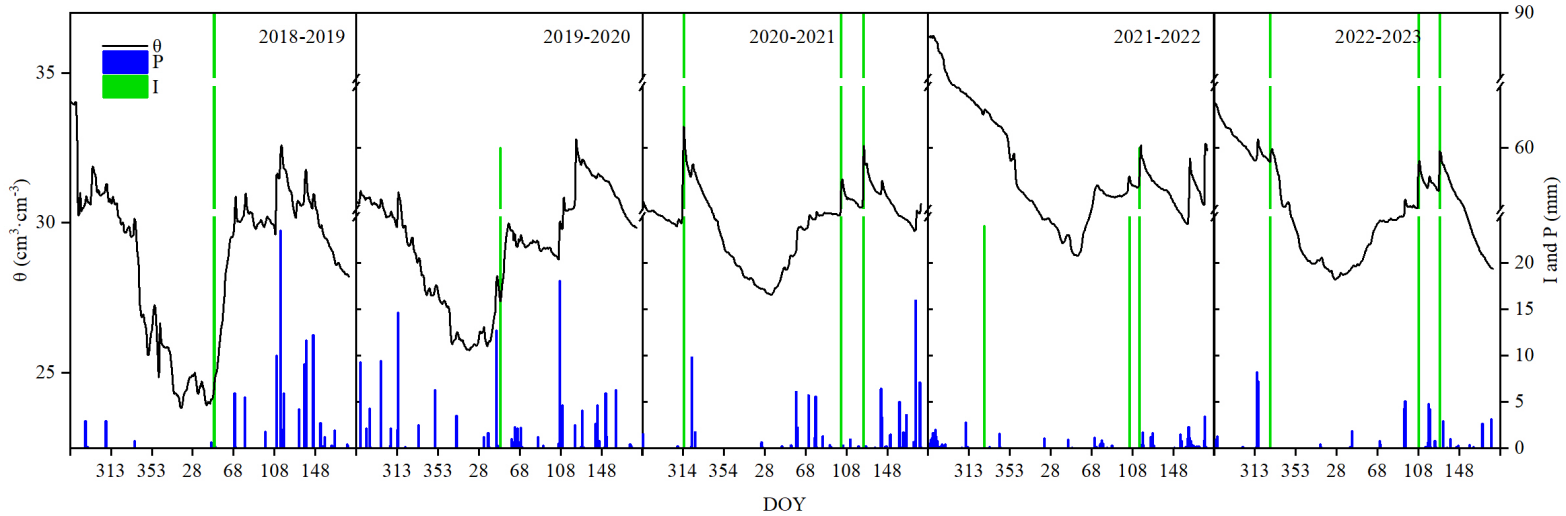
Winter wheat 2018–2023 water conditions, including soil volumetric moisture content (θ), irrigation (*I*), and rainfall (*P*).

The interannual variation of water conditions during the 5-year growth period of winter wheat is shown in **Figure 3**. The weather was typically semiarid, and precipitation varied significantly each year, with values of 107.8, 135.6, 84.3, 41.7, and 54.9 mm, over the five growing cycles. Compared with the long-term mean precipitation of 96.4 mm, the years fell into the categories of normal toward wet (2018–2019), nearly wet (2019–2020), normal toward dry (2020–2021), and extremely dry (2021–2023). The soil moisture (θ) within 1 m varied from 23.8 to 36.2 cm^3^ cm^−3^, averaging 30.4 cm^3^ cm^−3^ over the five seasons, primarily driven by irrigation and rainfall. Irrigation varied across experimental years, i.e., one irrigation of 70 mm at the active growing stage for the former two seasons (2018 to 2020) and three irrigations totaling 270 mm, respectively, at seedling (10 November 2020), jointing (13 April 2021), and flowering (5 May 2021) for the last three seasons (2020 to 2023).

**Figure 4:**
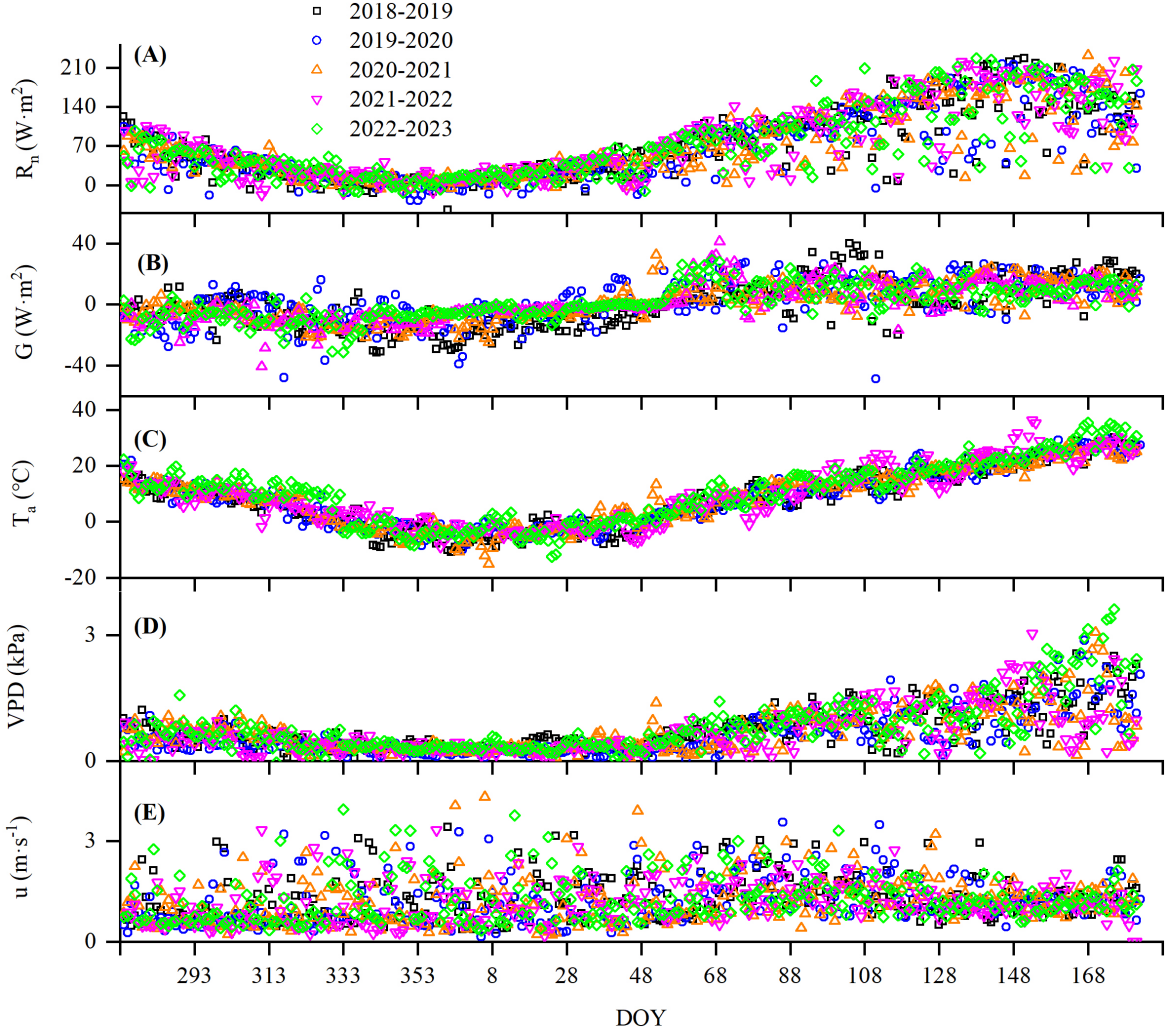
Changes in **(A)** net radiation (*R*_n_), **(B)** soil heat flux (*G*), **(C)** air temperature (*T*_a_), **(D)** vapor pressure difference (VPD), and **(E)** wind speed (*u*) in winter wheat measured by the Bowen ratio energy balance (BREB) system from 2018 to 2023. DOY means day of years.

The variation in meteorological factors during the 5-year growth period of winter wheat is shown in **Figure 4**. They showed a similar trend, decreasing first till January and then starting to increase thereafter. The 30-min daily mean *R*_n_ and *T*_a_ changed from −44 to 233 w m^−2^ and −15 to 36°C with a mean of 71 w m^−2^ and 8.9°C, respectively, during 2018–2023, all of which peaked in June (**Figures 4A, C**). The daily VPD varied from 0 to 3.6 kPa, averaged 0.7 kPa over the five seasons (**Figure 4D**), and turned flat in winter and started to rise in the greening period. The daily *G* and *u* ranged from −48 to 41 w m^−2^ and from 0 to 4.3 m s^−1^, respectively, and averaged 0.83 w m^−2^ and 1.2 m s^−1^ over the five seasons (**Figures 4B, E**).

### 3.2 Comparison of daily ET

#### 3.2.1 *r*_c_ models with original parameters

**Figure 5:**
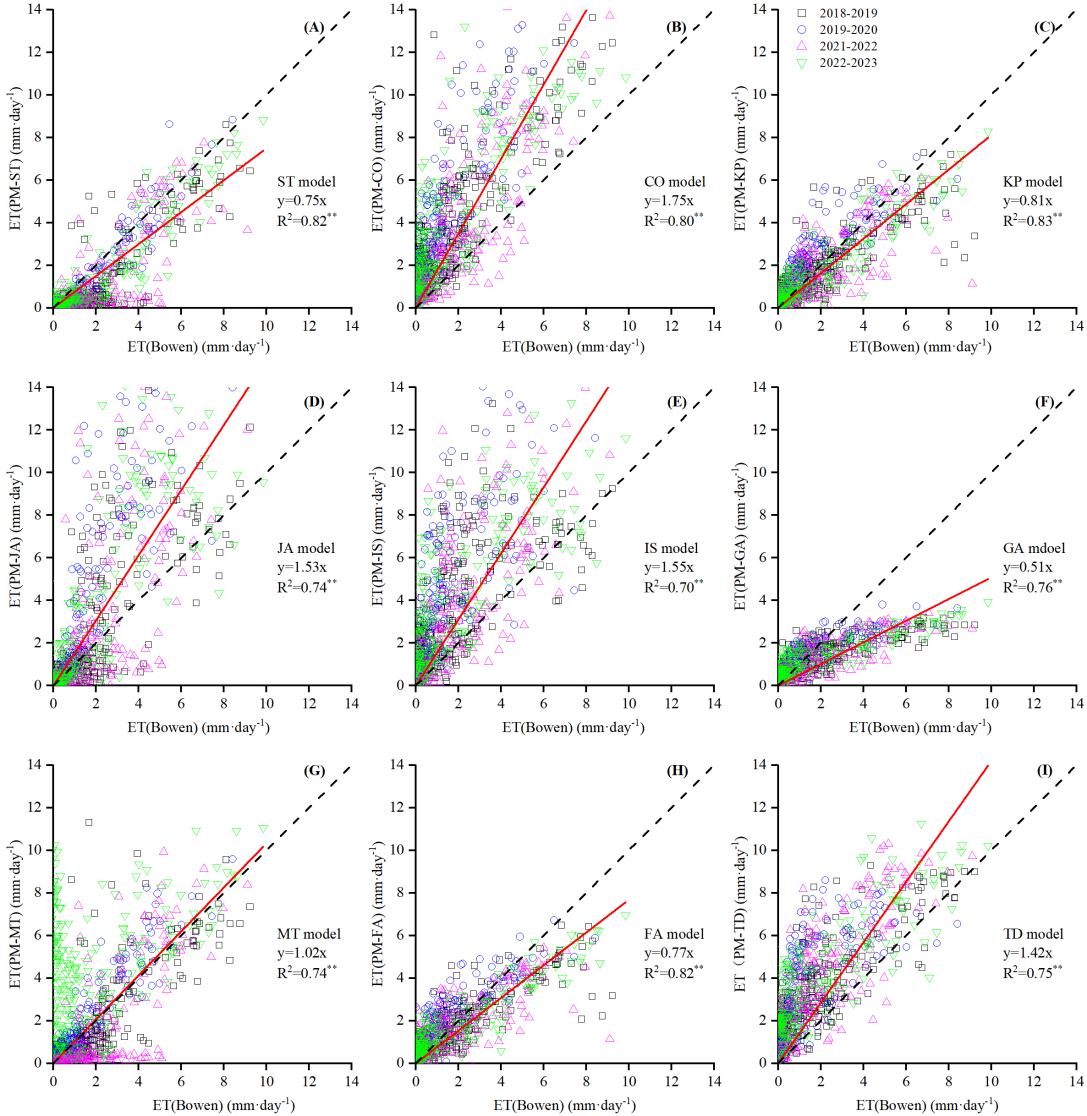
Scatter plot of daily ET (mm d^−1^) between nine PM-*r*_c_ models (**(A)** PM-ST, **(B)** PM-CO, **(C)** PM-KP, **(D)** PM-JA, **(E)** PM-IS, **(F)** PM-GA, **(G)** PM-MT, **(H)** PM-FA and **(I)** PM-TD model) with original parameter estimation against BREB measurement.

**Figure 6:**
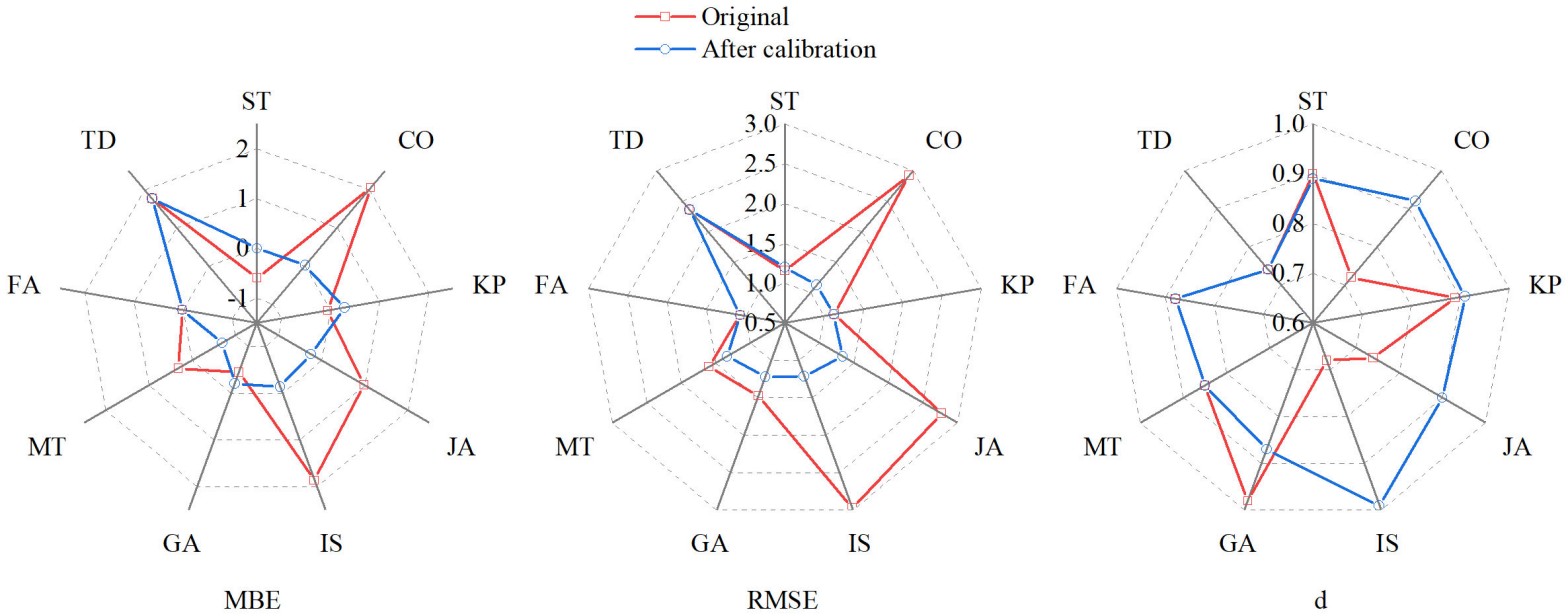
Comparison of the mean bias error (MBE, mm d^−1^), mean root square error (RMSE, mm d^−1^), and index of agreement (*d*) of winter wheat daily ET by different *r*_c_ models with original parameters and after parameter calibration.

The scatter plot of daily ET in **Figure 5** showed a significant correlation (*p* < 0.001) between the PM estimated from nine *r*_c_ models with original parameters and the BREB measured values. The *R* ^2^ values ranged from 0.7 to 0.83, with the PM-KP obtaining the highest correlation and the PM-IS being the lowest. However, the slope of the linear relationships varied significantly, ranging from 0.51 to 1.75 with the PM-MT model being closest to 1. Clearly, four *r*_c_ models (the PM-ST, PM-KP, PM-GA, and PM-FA) underestimated daily ET, as indicated by their regression slopes (0.51–0.81) of less than 1 (**Figures 5A, C, F, H**). This was also reflected in their daily mean difference (MBE), ranging from −0.59 to 0.02 mm d^−1^ (**Figure 6**), with the PM-FA having the largest value and the PM-ST having the smallest. The PM-GA had the largest underestimation at only half of the true value. An MBE of less than 0 meant underestimation and *vice versa*. However, the MBE for the FA model was greater than 0, likely because it generally overestimated ET when the daily ET was less than 2 mm d^−1^ (e.g., sparse vegetation cover) (Li et al., 2015). The index of agreement (*d*) of these *r*_c_ models was greater than 0.85 (**Figure 6**), with the PM-GA having the highest value and the PM-FA having the lowest. The RMSE values for these four *r*_c_ models ranged from 1.07 to 1.47 mm d^−1^, averaging 1.21 mm d^−1^ (**Figure 6**), suggesting a performance order of PM-FA > PM-KP > PM-ST > PM-GA. Notably, the PM-KP performed similarly to the PM-FA, producing values of slope, *R* ^2^, MBE, RMSE, and *d* within 0.04, 0.01, 0.08 mm d^−1^, 0.06 mm d^−1^, and 0.01 of each other.

Five *r*_c_ models, i.e., the PM-CO, PM-JA, PM-IS, PM-MT, and PM-TD, overestimated daily ET, as indicated by their regression slope (**Figures 5B, D, E, G, I**) of larger than 1. The slope of the PM-MT was closest to 1 (1.02), and that of the others were greater than 1.4. Meanwhile, the PM-MT obtained the smallest MBE at 0.32 mm d^−1^, while the others ranged from 0.98 to 2.05 mm d^−1^, averaging 1.33 mm d^−1^ (**Figure 6**). The PM-CO model had the largest overestimation. The *d* values of these models were lower than those of the underestimated models, ranging from 0.68 to 0.85 with an average of 0.77. Among these, PM-IS performed the worst and PM-TD performed the best. The RMSE values for the overestimated models ranged from 1.6 to 2.97 mm d^−1^, averaging 2.52 mm d^−1^. Performance decreased in the following order: PM-MT > PM-TD > PM-JA > PM-CO > PM-IS (**Figure 6**).

The accuracy of PM-ST, PM-KP, and PM-FA models without parameterization was acceptable in this study. The PM-KP model performed aligning with the estimation results of Yan et al. (2020) for winter wheat ET in humid regions. However, the RMSE increased by 38.3% compared to their value of 0.81 mm d^−1^. The FA model did not require parameter correction and needed fewer meteorological factors, making it suitable for widespread application. Its simulation results differed from those of Li et al. (2015) for maize and grape under partial and dense canopy stages, where their RMSE was much high at 7 mm d^−1^. The ST model, a JA-type model, ranked behind the KP and FA models, requiring LAI values during the calculation process. Having more parameters and complex calculation processes did not improve the accuracy.

The other six *r*_c_ models (CO, JA, IS, GA, MT, and TD) presented great error (RMSE > 1.5 mm d^−1^) without parameterization (**Figure 6**). Historically, the MT model was the most widely used in PM equation (*r*_c_ =70 s m^−1^) to estimate grassland ET (Gharsallah et al., 2013). The MT model performed best in regression slope but significantly overestimated in 2022–2023 and underestimated in 2021–2022, resulting in a 4-year total slope of 1.02 (**Figure 5G**). This inconsistency may be due to *r*_c_ being assigned a fixed value that did not match the actual situation. The TD model, which did not require parameter correction, showed good results in Liu et al. (2011) and Yan et al. (2020) with RMSE values of 0.79 and 0.85 mm d^−1^, respectively. They were much lower than those in our study. This discrepancy may be because these studies used only daytime data, whereas using data from the entire day can introduce uncertainty in daily ET. When VPD and *R*_n_-G were less or near zero, and when *r*_I_ and r* in the TD model had negative or uncertain values, *X* had no solution (the solution of the TD method equation, **Table 1**). Thus, the TD model was only applicable when *r*_I_ was precise (Perez et al., 2005). In another study, Katerji et al. (2011) compared the KP and TD models over four crops and found that the TD model’s overestimation was attributed to the theoretical limitations, neglecting the effect of aerodynamic resistance. The inability of ST, CO, JA, IS, and GA *r*_c_ models to estimate daily ET with original parameters was due to the inadequacy of these parameters, attributed to differences in crop types and significant variations in environmental factors, especially regional climatic water conditions (Forster et al., 2022). Therefore, using these models to estimate ET required parameter calculation.

#### 3.2.2 *r*_c_ models with calibrated parameters

**Figure 7:**
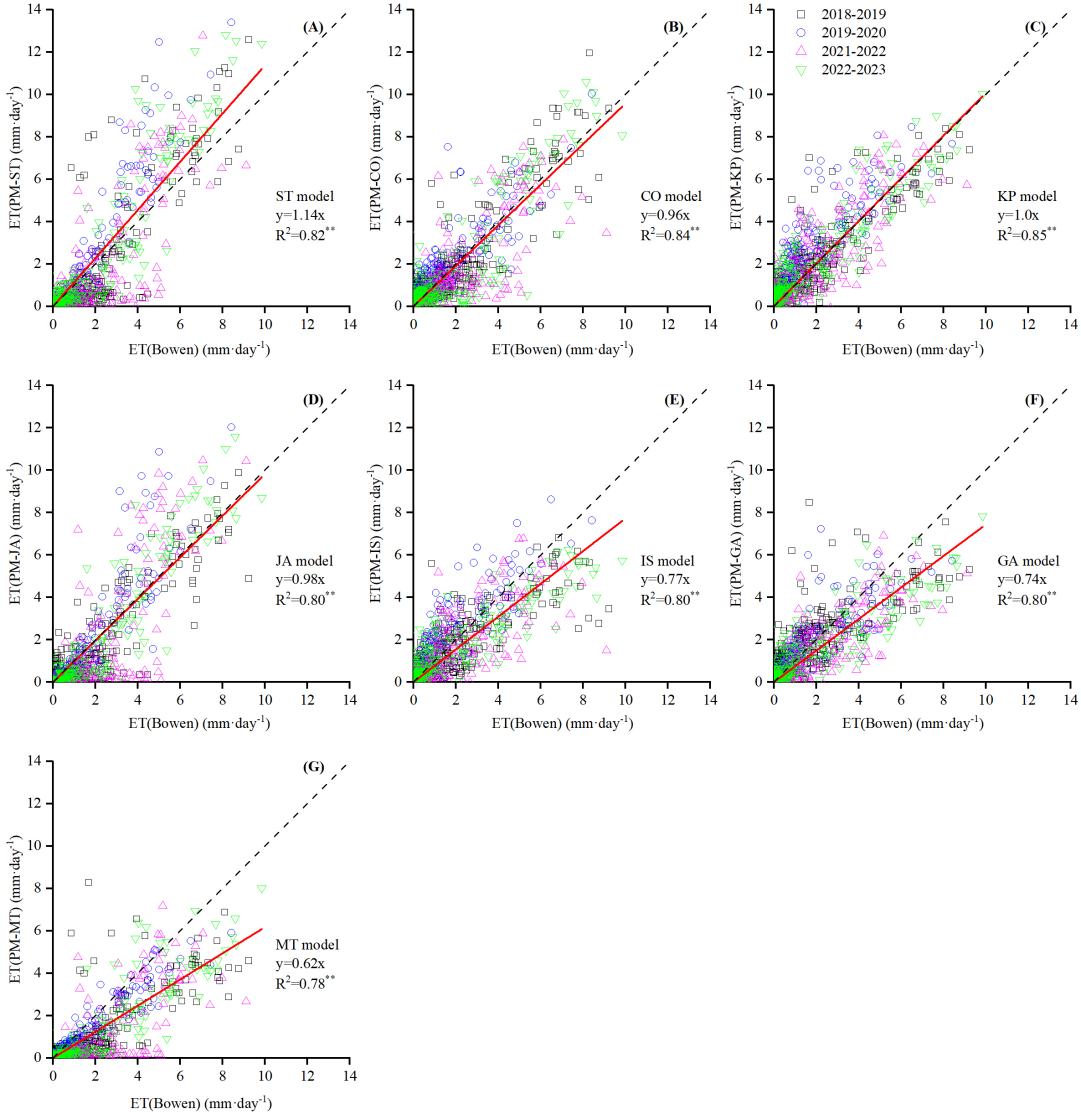
Scatter plot of daily ET (mm d^−1^) between seven PM-*r*_c_ models (**(A)** PM-ST, **(B)** PM-CO, **(C)** PM-KP, **(D)** PM-JA, **(E)** PM-IS, **(F)** PM-GA and **(G)** PM-MT model) after calibration estimation against BREB measurement (except for FA and TD models).

The scatter plot of the daily ET estimated by the PM equation with seven *r*_c_ models (excluding the FA and TD) with calibrated parameters against BREB measurement is shown in **Figure 7**. It could be clearly seen that scatter plots of *r*_c_ models were closer to the 1:1 line after calibration, indicating a significant correlation between measured values. Compared to using original parameters, seven *r*_c_ models (except the FA and TD) performed better after recalibration. *d* increased from 0.69–0.93 to 0.86–0.93 with the average *d* value increasing by 13.8%. *R* ^2^ increased from 0.7–0.83 to 0.78–0.85, with the average *R* ^2^ increasing by 19%, indicating improved stability and reliability of the *r*_c_ models. The PM-KP obtained the highest correlation, while the PM-MT had the lowest.

Five *r*_c_ models (PM-CO, PM-JA, PM-IS, PM-GA, and PM-MT) underestimated the daily ET values, with the regression slopes ranging from 0.62 to 0.96, averaging 0.81 (**Figures 7B, D–G**). MBE values ranged from −0.70 to 0.01 mm d^−1^, averaging −0.26 mm d^−1^, with the PM-MT having the largest underestimation and the PM-CO having the smallest (**Figure 6**). The underestimated *r*_c_ models produced RMSE values ranging from 1.12 to 1.34 mm d^−1^, averaging 1.25 mm d^−1^ (**Figure 6**), according to their RMSE ranked as PM-CO > IS > GA > JA > MT. The RMSE values were 16.25%–91.64% lower than using the original parameters, averaging 41.16%, indicating that the accuracy of *r*_c_ models has been improved.

PM-ST and PM-KP showed a light trend of overestimation with regression slopes of 1.14 and 1, respectively (**Figures 7A, C**). The PM-ST model obtained a better MBE value, while the PM-KP model performed better in terms of RMSE. Their MBEs were 0.00 and 0.29 mm d^−1^, and the RMSE values were 1.20 and 1.12 mm d^−1^, respectively (**Figure 6**). It was worth noting that the RMSE of PM-ST model increased by 3.4%, while the PM-KP model only decreased RMSE by 0.88%, suggesting that the accuracy has not improved.

As described above, the performance of the nine *r*_c_ models, based on RMSE values (**Figure 6**), was ranked as PM-FA > CO > KP > ST > IS > GA > JA > MT > TD. The performance of the first eight models was acceptable. Although parameter recalibration could reduce the RMSE value of the *r*_c_ model, they were still higher than that of the PM-FA model. However, this process significantly improved the regression slope of the models. The FA model’s RMSE still performed better than others after parameter correction. Ortega-Farias et al. (2004) and Ortega-Farias et al. (2006) also indicated that the PM-FA model accurately estimated ET for soybean and tomato. However, the regression slope of the PM-FA was the second to last among the eight acceptable models, possibly because it was an empirical method and failed to consider the effect of water stress condition. Unlike with the previous research (Li et al., 2015), the FA model systematically overestimated maize and grapevine ET, with RMSE exceeding 7 mm d^−1^ during both low and high LAI stages. This overestimation was primarily due to the underestimating canopy resistance, particularly during the sparse canopy stage. While the model accounted physiological control on resistance, it failed to consider the restrictive effects of soil.

The PM-CO, IS, and JA *r*_c_ models exhibited significant errors before parameterization but achieved satisfactory accuracy afterward (**Figures 5**–**7**). The PM-CO simulated maize and vineyards more accurately than in Li et al. (2015), with an RMSE 31.3% lower than theirs. Although the PM-CO model had good accuracy, it involved the most complicated calculation process among all *r*_c_ models and required the most meteorological parameters. The difficulty of obtaining these data should be considered in practical applications. The successful application of the PM-IS model in this study was consistent with Howell et al. (1997) for wheat, with an RMSE reduction of 18.3%. However, the RMSE for corn and sorghum increased by 29.2% and 57.5%, respectively. This may indicate that the PM-IS model was more suitable for estimating ET of crops with higher plant height and greater canopy temperature differences.

Compared with Zhang et al. (2008) and Li et al. (2015), the accuracy of the PM-JA model has improved, but it was still inferior to other models and ranked last among all acceptable models. Zhang et al. (2008) indicated that the PM-JA model overestimated the vineyard daily ET in an arid desert region of northwest China and was inaccurate after rainfall. The JA model presented uncertain results during the partial canopy stage but simulated ET accurately under the full canopy (Li et al., 2015). This may explain the larger overall RMSE, which also showed that the accuracy of model estimation was not related to the model’s complexity.

For both the PM-KP and ST models, parameterization appeared to be unnecessary. The reduction in RMSE after calibrating the KP model calibration was not negligible, with only a 0.01 difference before and after calibration. This indicated that parameter recalibration did not enhance the KP model’s accuracy, though it did achieve an optimal linear regression slope (value = 1), consistent with Rana et al. (2012). Compared to the simulations of tomato, maize, canola, and tea by Rana et al. (2012); Srivastava et al. (2018); Liu et al. (2012), and Zhang et al. (2022a), the RMSE increased by 43.8%, 66.9%, −3.6%, and 8.9%, respectively, indicating that parameter calibration did not improve accuracy. The PM-ST model’s RMSE was identical to that reported by Xing et al. (2024) for the kiwifruit. However, parameter correction did not improve its accuracy; instead, it slightly increased the RMSE. Nevertheless, the regression slope, *d*, and MBE values were improved. This may be due to significant annual variations, with 2 years of overestimation and 2 years of underestimation, leading to a slight increase in RMSE.

The PM-GA and MT models still exhibited some significant errors even after parameter calibration, but their results were better than those of the TD model, which did not require correction. In the GA model, the maximum stomatal conductance was set as a constant value. However, in the natural environment, this parameter dynamically fluctuated in response to climatic variations. This discrepancy may contribute to the observed significant underestimation of the GA model (García-Santos et al., 2009; Xu et al., 2021). Similar to the GA model, the MT model also underestimated ET due to its overestimation of *r*_c_ and the uncertainty of nighttime *r*_c_. Therefore, these two models should be used with caution.

Although the PM-FA, KP, and ST models can estimate daily ET of winter wheat regardless of whether they were calibrated or not, they each had limitations. Without parameterization, these three models noticeably underestimated daily ET. In this study, calibrating the KP and ST models appeared redundant, as the RMSE of the KP model decreased by only 0.01 mm d^−1^, while that of the ST model increased by 0.04 mm d^−1^ after calibration. In comparison, the errors did not improve and even worsened in some cases. Based on the results of Yan et al. (2020), the KP model, originally applied in humid conditions, can still be effectively used under the semiarid conditions of this study. Additionally, the PM-CO, IS, ST, GA, JA, and MT models, similar to previous studies, required parameter calibration before they were used to estimate ET. However, these models required more parameters or meteorological data compared to the KP model. The FA model did not require parameterization, which often demanded extensive soil moisture data, making it useful when parameter calibration was not feasible without measured ET values.

### 3.3 Comparison of seasonal ET

#### 3.3.1 *r*_c_ models with original parameters

In order to analyze the seasonal cumulative ET of winter wheat simulated by PM with different *r*_c_ models, this study divided the entire growth period of winter wheat into three stages: the seeding period in October and November, the wintering period from December to February of the following year, and the rapid growth period from March to June. The seasonal ET of winter wheat during the seeding, wintering, and rapid growth periods over four growth years, observed using the BERB method, ranged as follows: 50.07 to 98.35 mm with an average of 79.42 mm for the seeding period, 12.56 to 44.2 mm with an average of 27.05 mm for the wintering period, and 251.8 to 409.7 mm with an average of 359.34 mm for the active growing period. Throughout the entire reproductive period, the proportion of total ET was 10.6% to 25.69% during the seeding period, with an average of 17.37%; 2.66% to 9.10% during the wintering period, with an average of 5.91%; and 65.80% to 86.74% during the rapid growth period, with an average of 76.72%.

**Figure 8:**
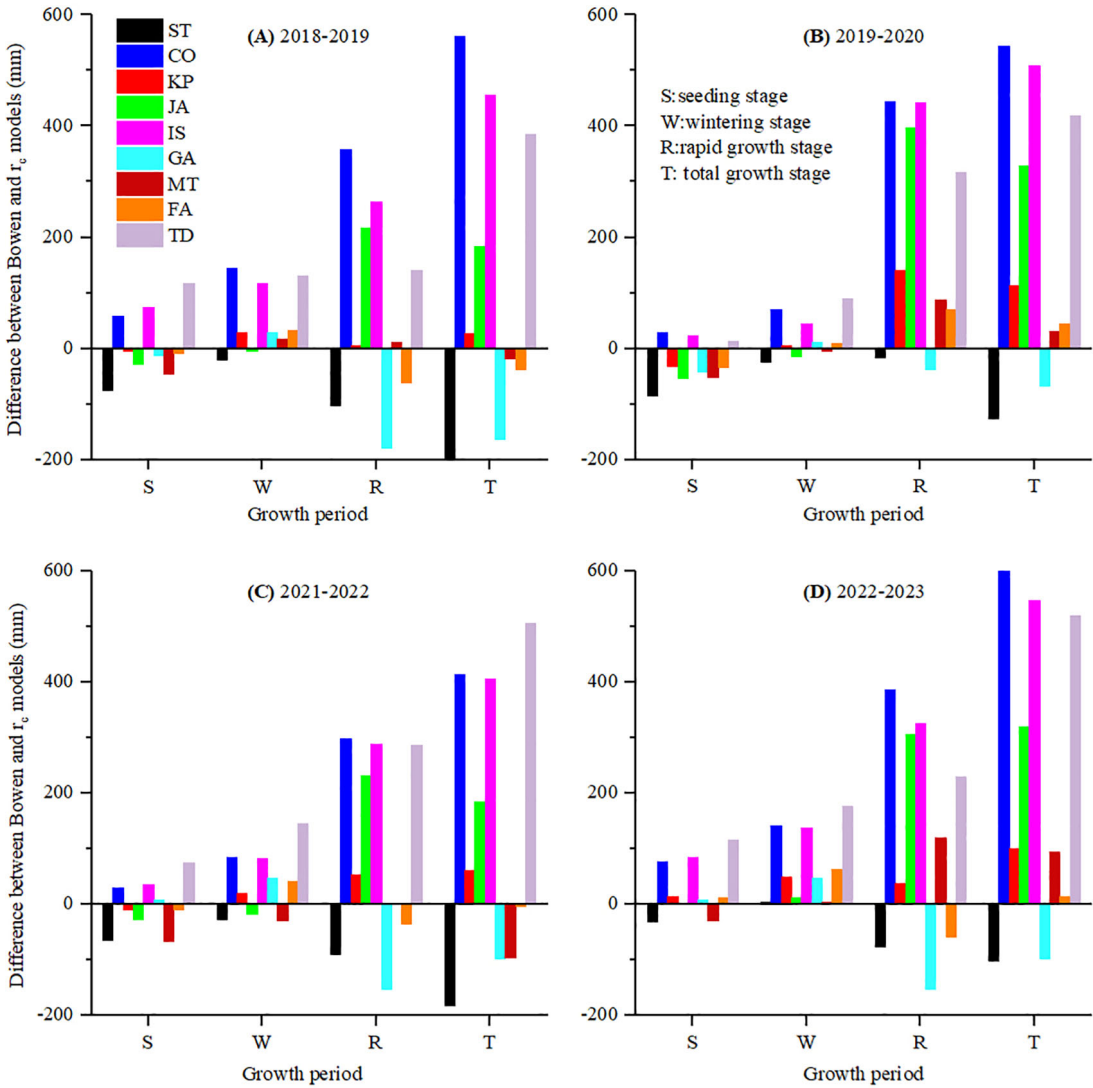
The difference between the seasonal ET measured by BREB and estimated by the PM equation combined with nine *r*_c_ models of winter wheat using original parameters in **(A)** 2018-2019, **(B)** 2019-2020, **(C)** 2021-2022 and **(D)** 2022-2023 growth season.

**Figure 9:**
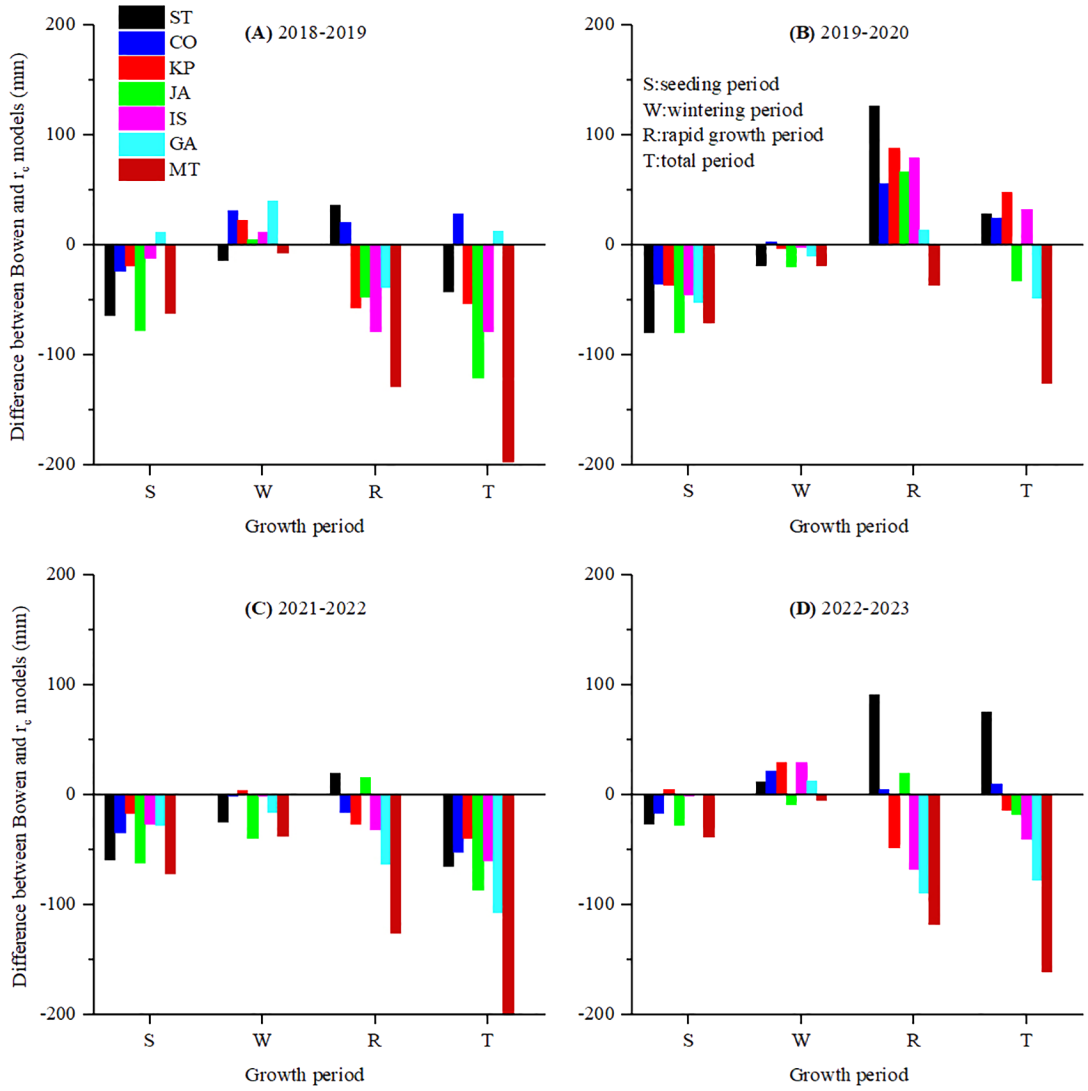
The difference between the seasonal ET measured by BREB and estimated by the PM equation combined with seven *r*_c_ models of winter wheat after parameter calibration (except for FA and TD models) in **(A)** 2018-2019, **(B)** 2019-2020, **(C)** 2021-2022 and **(D)** 2022-2023 growth season.

To compare winter wheat seasonal ET across different growth stages, the differences between BREB observations and PM combined *r*_c_ model estimations over 4 years are shown in **Figures 8** and **9**. Over the entire growth period, the PM-ST and PM-GA *r*_c_ models consistently underestimated seasonal ET. The total ET differences between the two *r*_c_ models applied in PM and the BREB measurements varied from −197.12 to 12.27 mm, with an average of −103.59 mm (**Figure 8**). The MBE and RMSE for the PM-ST and GA models were −152.06 and −115.79 mm, and 157.05 and 121.23 mm, respectively (**Table 2**). The other seven *r*_c_ models (i.e., PM-CO, KP, JA, IS, MT, FA, and TD) overestimated seasonal ET. The differences in ET between these seven *r*_c_ models applied in PM and the ET determined by the BREB ranged from −95.33 to 603.52 mm, with an average of 257.65 mm (**Figure 8**). The MBE values varied from 2.83 to 530.47 mm (**Table 2**), with the PM-MT model performing the best and the PM-CO model performing the worst. The RMSE values ranged from 29.79 to 535.26 mm (**Table 2**), based on performance that decreased according to FA > MT > KP > JA > TD > IS > CO.

**Table 2:** Comparison of the mean bias error (MBE, mm) and root mean square error (RMSE, mm) of seasonal accumulated ET of winter wheat by different *r*_c_ models with original parameters and after parameter calibration at different growth stages.

Model	Stage	Original parameter				Parameter calibration			
		Seeding period	Wintering period	Rapid growth period	Total ET	Seeding period	Wintering period	Rapid growth period	Total ET
ST	MBE	−63.88	−16.87	−71.31	−152.06	−57.44	−11.69	68.00	−1.13
	RMSE	67.05	21.25	78.44	157.05	60.56	18.01	80.41	55.92
CO	MBE	48.64	110.22	371.60	530.47	−27.59	13.65	16.31	2.36
	RMSE	52.57	115.17	375.27	535.26	28.59	19.22	30.96	32.18
KP	MBE	−8.64	25.46	58.50	75.32	−16.76	12.98	−10.96	−14.74
	RMSE	18.63	29.85	77.01	82.38	22.30	18.49	59.20	41.68
JA	MBE	−26.87	−6.48	287.39	254.03	−61.80	−15.97	13.40	−64.37
	RMSE	33.46	12.98	296.26	263.54	65.15	22.81	42.67	76.35
IS	MBE	54.51	95.02	329.71	479.25	−21.34	9.47	−24.73	−36.59
	RMSE	60.13	101.40	336.75	482.23	27.06	15.76	67.38	55.60
GA	MBE	−17.51	24.82	−123.10	−115.79	−17.37	6.70	−44.43	−55.11
	RMSE	25.29	28.40	134.05	121.23	30.07	22.98	58.57	70.58
MT	MBE	−48.12	−3.68	54.62	2.83	−60.83	−17.00	−102.10	−179.93
	RMSE	49.88	17.47	74.36	69.63	62.27	21.41	109.08	184.50
FA	MBE	−10.16	36.18	−21.80	4.22	–	–	–	–
	RMSE	19.01	41.04	57.93	29.79	–	–	–	–
TD	MBE	79.33	135.47	242.68	457.48	–	–	–	–
	RMSE	89.75	139.04	251.79	460.98	–	–	–	–

During the seeding period, the PM-ST, KP, JA GA, MT, and FA models underestimated ET, with MBE ranging from −63.88 to −8.64 mm (averaging −29.19 mm) and RMSE ranging from 18.63 to 67.05 mm (averaging 35.55 mm). The PM-CO, IS, and TD models overestimated ET, with MBE values of 48.64, 84.51, and 79.33 mm, and RMSE values of 52.57, 60.13 and 89.75 mm, respectively (**Table 2**). The average RMSE values during the seeding period were nearly half of the measured ET. This funding was similar to that of Sun et al. (2007), who suggested that *r*_c_ models showed large errors during the sparse canopy stage or when the LAI was less than 2. However, in this study, the errors were even larger.

During the wintering period, when ET was at its lowest, the ST, JA, and MT models underestimated ET, while the other models overestimated it. The MBE values ranged from −3.68 to 135.47 mm, with an average of 44.46 mm, and the RMSE values ranged from 12.98 to 139.04 mm, with an average of 56.29 mm (**Table 2**). Although these average values were larger than the measured values, the accuracy of the *r*_c_ models without parameter calibration did not rely heavily on this stage, as ET during this period accounted for only an average of 5.91% of the total ET, which was consistent with the findings of Gharsallah et al. (2013).

All *r*_c_ models recorded their highest RMSE during the rapid growth period, ranging from 57.93 to 375.27 mm, with an average of 186.87 mm. Among these, the FA model performed best and the CO model performed worst, indicating that the rapid growth period was the stage with the least accuracy in estimating ET. Over- or underestimation at this stage did not determine the overall ET estimation trend, as seen with the MT model. The ST, FA, and GA models underestimated ET during this period, with MBE values of −71.31, −21.80, and −123.10 mm, respectively. Among these, the FA model performed the best and the GA performed the worst. The other six *r*_c_ models overestimated ET, with MBE values ranging from 54.62 to 371.6 mm (**Table 2**).

Without parameter calibration, the PM-FA, PM-MT, and PM-KP models were acceptable on a seasonal scale, mainly due to their excellent performance during the rapid growth period. They exhibited both over- and underestimation across the three growth periods, resulting in the total ET errors offsetting each other. The FA model performed perfectly in all years (**Figure 8**) and achieved the best RMSE values (**Table 2**). The FA model relied on the climatic factors and soil water, which was a function of soil moisture and *r*_i_, and was defined as the water vapor transfer from the soil and plants to the atmosphere (Chen et al., 2022). Due to the inclusion of the moisture content factor, it better reflected the early stage of soil ET. The RMSE of the KP and MT models were acceptable; however, the differences between KP model and BREB for the 2019–2020 and 2022–2023 periods were 112.71 and 99.35 mm, respectively. Additionally, the differences between the MT model and BREB for the 2021–2022 and 2022–2023 periods were −95.33 and 95.11 mm, respectively. These discrepancies seemed unacceptable for practical estimation applications (**Figure 8**). Therefore, the KP and MT models without calibration should be used with caution in seasonal ET assessments.

It was noteworthy that the MT model was rejected on the uncalibrated daily scale, while the ST was accepted on the daily scale but rejected on the seasonal scale. This suggested that the *r*_c_ model, which estimated ET on the daily scale, may not be suitable for estimating seasonal ET, as the same model performed differently at different scales (Howell et al., 1997). This phenomenon may be attributed to variations in climate conditions across years and the differing performance of models at various growth stages of winter wheat. Interestingly, over- and underestimation at different stages can offset each other, improving the overall reproductive cycle results. The MT model performed well on the seasonal scale because it underestimated seeding period daily ET and overestimated it during the rapid growth period (**Figure 8**), causing the total ET errors to cancel each other out. The ST model significantly underestimated the daily ET across all three growth stages (**Figure 8** and **Table 2**). The accumulation of these large errors resulted in total seasonal ET estimation, indicating that consistent estimation trends across growth stages can lead to larger errors in total ET.

The rejection of the six unacceptable models (i.e., ST, CO, JA, IS, GA, and TD) was primarily due to their poor performance during the rapid growth period (**Figure 8** and **Table 2**). Unlike in previous studies, Liu et al. (2011); Chen et al. (2022); Xing et al. (2024); Li et al. (2015); García-Santos et al. (2009), and Xing et al. (2024), the TD, JA, IS, and CO models significantly overestimated ET during this stage, while the GA and ST models significantly underestimated it. All of the unacceptable models exhibited the worst accuracy during the rapid growth stage, with severe overestimation or underestimation sometimes exceeding twice the ET value at this stage (**Table 2**). This indicated that the accuracy of seasonal ET estimation primarily depended on this period.

#### 3.3.2 *r*_c_ models with calibrated parameters

After parameter calibration, six *r*_c_ models (i.e., ST, KP, JA, IS, GA, and MT) underestimated the total seasonal ET. The total ET differences between these models and the direct determination method ranged from −235.31 to 74.88 mm, with an average of −68.76 mm. Among them, the ST model obtained the best average value, while the MT model had the worst (**Figure 9**). The MBE values varied from −179.93 to −1.13 mm (**Table 2**), with the ST model performing the best and the MT model performing the worst. The positive and negative MBE values on the seasonal scale were entirely consistent with the regression slope. Unlike on the daily scale, there was no instance where the MBE was greater than 0 but the slope was less than 1. The RMSE values ranged from 41.68 to 184.50 mm, with an average of 80.77 mm (**Table 2**). The models ranked by RMSE were as follows: KP > IS > ST > GA > JA > MT. Only the CO model generally overestimated the seasonal total ET, with differences from the BREB method ranging from −51.84 to 28.11 mm and an average of 2.36 mm (**Figure 9**). Its MBE value of 2.36 mm was second only to the ST model, and its RMSE of 32.18 mm was the best among all *r*_c_ models (**Table 2**).

Parameter calibration did not improve the accuracy of all *r*_c_ models across all growth stages. The RMSE values of the KP, JA, GA, and MT models during the seeding period, JA and MT models during the wintering period, and ST and MT models during the rapid growth period were 2.51%–94.74% higher than those of the uncorrected models. In contrast, the accuracy of the *r*_c_ models improved in other situations, with RMSE values decreasing by 9.69% to 91.75%. Except for the MT model, the MBE values for the three growth stages improved, indicating that the degree of overestimation or underestimation was reduced, resulting in more accurate estimation.

The ability of the *r*_c_ model to estimate seasonal ET depended on the simulation results during the rapid growth period. Six *r*_c_ models were accepted, with only the MT model being refused based on the RMSE values. The performance of the *r*_c_ models was clearly better than before, with the RMSE value reduced by 68.18%, indicating that the recalibration process significantly improved the accuracy of the *r*_c_ models, except for the MT model (**Figure 9** and **Table 2**). This improvement was attributed to better estimation results during the rapid growth period compared to previous results (Li et al., 2014). It should be noted that the underestimation occurred during the seeding stage, and the estimation error during this stage was primarily attributed to significant heterogeneity in water vapor transport within the model (Li et al., 2015). Compared to the daily scale, the accuracy of estimating seasonal ET was higher, consistent with findings by Gharsallah et al. (2013). This may be because the high and low ET estimation canceled each other out over the long-term growth period, making the cumulative seasonal ET close to the measured value (Irmak and Irmak, 2008).

The CO model showed better simulation results in the early stages of growth. This improvement was primarily because the CO followed the resistance law of fluid transfer, coupling the resistance of plants and soil into the overall canopy resistance. It accounted for the combined limiting effects of vegetation and soil on surface water transfer, and providing an accurate estimate of average surface resistance (Li et al., 2015). The FA model ranked second to the CO model. Although it still underestimated seasonal ET as it did on the daily scale, its results were better than those for daily ET estimation.

During the calibration of KP and IS model parameters, VPD had the greatest influence, making it the primary factor affecting the model error. However, when the VPD value was less than 2 kPa, the model error was minimal (Yan et al., 2020). During the seeding and wintering period, the VPD was generally below 2, resulting in better simulation results for these two models.

The GA model was rejected on the daily scale but accepted on the seasonal scale. As a function of *R*_n_ and VPD, it performed well during the seeding and wintering periods, and its results for the rapid growth period were also acceptable. Therefore, it can be used to estimate seasonal ET.

The accuracy of the ST model improved during the seeding and the wintering periods. However, its RMSE value increased during the rapid growth period, shifting from underestimation to overestimation. The combined effects of the seeding and the wintering periods improved the overall results, leading to a 64.39% reduction in RMSE.

The ST, JA, and MT models had large errors in estimating ET during the seeding and wintering periods because they all belonged to the JA-type model, which upscaled leaf-level resistance to canopy-level resistance (Lhomme, 2001; Wei et al., 2013). However, during these two growth periods, the LAI value was less than 1, leading to significant underestimation of ET due to the exposed surface. The ST and JA models slightly overestimated ET during the rapid growth period, but the rapid development of winter wheat during this period led to higher ET. The slight overestimation during this period essentially offset the serious underestimation during the seeding and wintering periods, resulting in a slight overestimation in the 4-year daily scale regression, but a more accurate simulation of the seasonal accumulation. Compared with the ET simulated by the JA-type *r*_c_ models used by Liu et al. (2020) in the seeding period, wintering period, green period, and maturity period, MBE and RMSE increased.

The MT model consistently underestimated ET across all three growth periods, while the TD model consistently overestimated it, leading to significant overall errors. Therefore, these two models were not suitable for studying winter wheat water consumption in this region at seasonal scale. Notably, the MT method was rejected after calibration due to its significant underestimation of ET during the seeding and rapid growth periods. During the early stages of crop development, the soil surface was nearly bare, and soil evaporation dominated the entire ET process. The assumption of the large leaf model led to significant errors (Wu et al., 2022). The significant overestimation by the TD model may be due to its sensitivity to VPD values, particularly when VPD ranged from 1.5 to 4 kPa. Additionally, research has shown that the TD model cannot be reliably applied at night (Yan et al., 2022).

In summary, after calibration, only the CO model outperformed the FA model, while the other models still performed worse. However, when comparing the calculation processes, it was clear that the FA model was much simpler than the CO model and did not require parameter calibration. The FA model was the most suitable method for seasonal ET estimation unless extremely high accuracy was required. When the FA and CO models lacked the necessary meteorological factor, the IS, GA, JA, ST, and KP models can be used to estimate seasonal ET based on known meteorological data.

## 4 Conclusions and recommendations

The parameter calibration process significantly improved the stability and reliability of *r*_c_ models in estimating ET on both daily and seasonal scales. After calibration, the average RMSE was reduced by 29.03% and 68.18%, respectively, with the *r*_c_ model showing greater accuracy in simulating ET on a seasonal scale compared to a daily scale.

The rapid growth period was the primary stage of winter wheat water consumption. Although overestimation or underestimation during this period did not solely determine the overall trend, the accuracy of *r*_c_ model estimation heavily depended on this period. The estimation of ET during the wintering period had little impact on overall accuracy. Underestimation of ET typically occurred during the seeding stage.

The simulation effects of nine canopy resistance models on winter wheat ET were examined. Among the models that did not require parameter calibration, the FA model provided accurate ET estimated on both daily and seasonal scales, while the TD model exhibited large errors and was not recommended. Without parameter calibration, the KP and ST models were suitable for daily scale use, while the KP and MT models were suitable for seasonal scale use. After calibration, the CO, KP, ST, IS, GA, JA, and MT can be used at the daily scale, while the CO, KP, IS, ST, GA, and JA were suitable for the seasonal scale (listed in order of increasing RMSE values). Model complexity did not directly correlate with the accuracy of ET estimation; a more complex model did not necessarily yield better results. Whether using original parameters or after calibration, the FA model consistently ranked in the top two and could be used in any scenario due to its simpler calculation process. It was recommended to select the most suitable model based on known meteorological data, model complexity, and simulation accuracy. This study demonstrated that the FA and KP models, after calibration, were recommended for estimating daily and seasonal ET in semiarid regions using the PM equation. The CO, GA, ST, IS, and JA models can also be considered as alternatives when sufficient meteorological data were available. Nonetheless, this study also presented some limitations. It did not thoroughly explore the relationship between *r*_c_ simulated by the canopy resistance model and *r*_c_ inferred from that measured by BREB. Additionally, further investigation was needed to understand the inaccuracies of the *r*_c_ models and identify the key factors influencing the accuracy of canopy resistance models.

## Data Availability

The original contributions presented in the study are included in the article/supplementary material. Further inquiries can be directed to the corresponding author.

## References

[B1] Allen R. G. Pereira L. S. Raes D. Smith M. (1998). Crop evapotranspiration-Guidelines for computing crop water requirements-FAO Irrigation and drainage paper 56. Fao Rome 300, D05109.

[B2] Bowen I. S. (1926). The ratio of heat losses by conduction and by evaporation from any water surface. Phys. Rev. 27, 779. doi: 10.1103/PhysRev.27.779

[B3] Chen X. Yu L. Cui N. Cai H. Jiang X. Liu C.. (2022). Modeling maize evapotranspiration using three types of canopy resistance models coupled with single-source and dual-source hypotheses—A comparative study in a semi-humid and drought-prone region. J. Hydrol. 614. doi: 10.1016/j.jhydrol.2022.128638

[B4] Forster M. A. Kim T. D. H. Kunz S. Abuseif M. Chulliparambil V. R. Srichandra J.. (2022). Phenology and canopy conductance limit the accuracy of 20 evapotranspiration models in predicting transpiration. Agric. For. Meteorol. 315. doi: 10.1016/j.agrformet.2022.108824

[B5] García-Santos G. Bruijnzeel L. A. Dolman A. J. (2009). Modelling canopy conductance under wet and dry conditions in a subtropical cloud forest. Agric. For. Meteorol. 149, 1565–1572. doi: 10.1016/j.agrformet.2009.03.008

[B6] Gharsallah O. Facchi A. Gandolfi C. (2013). Comparison of six evapotranspiration models for a surface irrigated maize agro-ecosystem in Northern Italy. Agric. Water Manage. 130, 119–130. doi: 10.1016/j.agwat.2013.08.009

[B7] Han X. Li Y. (2010). Study on the micrometeorological characteristics and energy balance of winter wheat canopy. Meteorol. Environ. Res. 1, 81–86. doi: 10.5555/20113140921

[B8] Howell T. Steiner J. Schneider A. Evett S. Tolk J. (1997). Seasonal and maximum daily evapotranspiration of irrigated winter wheat, sorghum and corn—Southern High Plains. Trans. ASAE 40, 623–634. doi: 10.13031/2013.21321

[B9] Idso S. B. (1983). Stomatal regulation of evaporation from well-watered plant canopies: a new synthesis. Agric. Meteorol. 29, 213–217. doi: 10.1016/0002-1571(83)90068-7

[B10] Irmak A. Irmak S. (2008). Reference and crop evapotranspiration in south central Nebraska. II: measurement and estimation of actual evapotranspiration for corn. J. Irrig. Drain. 134, 700–715. doi: 10.1061/(asce)0733-9437(2008)134:6(700

[B11] Irmak S. Mutiibwa D. (2010). On the dynamics of canopy resistance: Generalized linear estimation and relationships with primary micrometeorological variables. Water Resour. Res. 46, 1–20. doi: 10.1029/2009wr008484

[B12] Irmak S. Mutiibwa D. Payero J. Marek T. Porter D. (2013). Modeling soybean canopy resistance from micrometeorological and plant variables for estimating evapotranspiration using one-step Penman–Monteith approach. J. Hydrol. 507, 1–18. doi: 10.1016/j.jhydrol.2013.10.008

[B13] Katerji N. Rana G. Fahed S. (2011). Parameterizing canopy resistance using mechanistic and semi-empirical estimates of hourly evapotranspiration: critical evaluation for irrigated crops in the Mediterranean. Hydrol. Processes 25, 117–129. doi: 10.1002/hyp.7829

[B14] Lhomme J. P. (2001). Stomatal control of transpiration: Examination of the Jarvis-type representation of canopy resistance in relation to humidity. Water Resour. Res. 37, 689–699. doi: 10.1029/2000wr900324

[B15] Li S. Hao X. Du T. Tong L. Zhang J. Kang S. (2014). A coupled surface resistance model to estimate crop evapotranspiration in arid region of northwest China. Hydrol. Processes 28, 2312–2323. doi: 10.1002/hyp.9768

[B16] Li S. Zhang L. Kang S. Tong L. Du T. Hao X.. (2015). Comparison of several surface resistance models for estimating crop evapotranspiration over the entire growing season in arid regions. Agric. For. Meteorol. 208, 1–15. doi: 10.1016/j.agrformet.2015.04.002

[B17] Liu C. Cui N. Gong D. Hu X. Feng Y. (2020). Evaluation of seasonal evapotranspiration of winter wheat in humid region of East China using large-weighted lysimeter and three models. J. Hydrol. 590, 125388. doi: 10.1016/j.jhydrol.2020.125388

[B18] Liu G. Hafeez M. Liu Y. Xu D. (2011). A study on actual evapotranspiration estimation based on the Todorovic method. Ground Based Methods Multi Scale Hydrol. 343, 145–150.

[B19] Liu G. Liu Y. Hafeez M. Xu D. Vote C. (2012). Comparison of two methods to derive time series of actual evapotranspiration using eddy covariance measurements in the southeastern Australia. J. Hydrol. 454-455, 1–6. doi: 10.1016/j.jhydrol.2012.05.011

[B20] Liu X. Y. Mei X. R. Li Y. Z. Wang Q. S. Zhang Y. Q. Porter J. R. (2009). Variation in reference crop evapotranspiration caused by the Angstrom-Prescott coefficient: Locally calibrated versus the FAO recommended. Agric. Water Manage. 96, 1137–1145. doi: 10.1016/j.agwat.2009.03.005

[B21] López-Urrea R. Chávez J. (2019). One-step approach for estimating maize actual water use: part II. Lysimeter evaluation of variable surface resistance models. Irrigation Sci. 37, 123–137 doi: 10.1007/s00271-018-0607-7

[B22] Lovelli S. Perniola M. Arcieri M. (2008). Water use assessment in muskmelon by the Penman-Monteith “one-step” approach. Agric. Water Manage. 95, 1153–1160. doi: 10.1016/j.agwat.2008.04.013

[B23] Meng L. I. Chu R. H. Islam A. Jiang Y. L. Shen S. H. (2021). Estimating daily actual evapotranspiration of a rice–wheat rotation system in typical farmland in the Huai River Basin using a two-step model and two one-step models. J. Integr. Agric. 20, 274–288. doi: 10.1016/S2095-3119(20)63223-3

[B24] Monteith J. L. (1965). Evaporation and environment. Symp. Soc. Exp. Biol. 19, 205–234. 5321565

[B25] Niyogi D. Alfieri J. G. Blanken P. D. Chen F. LeMone M. A. Mitchell K. E.. (2008). Estimation of the minimum canopy resistance for croplands and grasslands using data from the 2002 international H2O project. Monthly Weather Rev. 136, 4452–4469. doi: 10.1175/2008mwr2524.1

[B26] Ortega-Farias S. Olioso A. Antonioletti R. Brisson N. (2004). Evaluation of the Penman-Monteith model for estimating soybean evapotranspiration. Irrigation Sci. 23, 1–9. doi: 10.1007/s00271-003-0087-1

[B27] Ortega-Farias S. O. Olioso A. Fuentes S. Valdes H. (2006). Latent heat flux over a furrow-irrigated tomato crop using Penman-Monteith equation with a variable surface canopy resistance. Agric. Water Manage. 82, 421–432. doi: 10.1016/j.agwat.2005.07.028

[B28] Penman H. L. (1948). Natural evaporation from open water, bare soil and grass. Proc. R. Soc. London Ser. A. Math. Phys. Sci. 193, 120–145. doi: 10.1098/rspa.1948.0037 18865817

[B29] Perez P. J. Lecina S. Castellvi F. Martínez-Cob A. Villalobos F. J. (2005). A simple parameterization of bulk canopy resistance from climatic variables for estimating hourly evapotranspiration. Hydrol. Processes 20, 515–532. doi: 10.1002/hyp.5919

[B30] Perrier A. (1975). Etude physique de l’évapotranspiration dans les conditions naturelles. III. Evapotranspiration réelle potentielle Des. couverts végétaux. 26, 229–243.

[B31] Qiu R. Liu C. Cui N. Wu Y. Wang Z. Li G. (2019). Evapotranspiration estimation using a modified Priestley-Taylor model in a rice-wheat rotation system. Agric. Water Manage. 224. doi: 10.1016/j.agwat.2019.105755

[B32] Rana G. Katerji N. Lazzara P. Ferrara R. M. (2012). Operational determination of daily actual evapotranspiration of irrigated tomato crops under Mediterranean conditions by one-step and two-step models: Multiannual and local evaluations. Agric. Water Manage. 115, 285–296. doi: 10.1016/j.agwat.2012.09.015

[B33] Shahrokhnia M. H. Sepaskhah A. R. (2012). Evaluation of wheat and maize evapotranspiration determination by direct use of the Penman–Monteith equation in a semi-arid region. Arch. Agron. Soil Sci. 58, 1283–1302. doi: 10.1080/03650340.2011.584216

[B34] Shen Y. Kondoh A. Tang C. Zhang Y. Chen J. Li W.. (2002). Measurement and analysis of evapotranspiration and surface conductance of a wheat canopy. Hydrol. Processes 16, 2173–2187. doi: 10.1002/hyp.1149

[B35] Spank U. Köstner B. Moderow U. Grünwald T. Bernhofer C. (2016). Surface conductance of five different crops based on 10 years of Eddy-covariance measurements. Meteorol. Z. 25, 251–266. doi: 10.1127/metz/2016/0732

[B36] Srivastava R. K. Panda R. K. Chakraborty A. Halder D. (2018). Comparison of actual evapotranspiration of irrigated maize in a sub-humid region using four different canopy resistance based approaches. Agric. Water Manage. 202, 156–165. doi: 10.1016/j.agwat.2018.02.021

[B37] Stannard D. I. (1993). Comparison of Penman-Monteith, Shuttleworth-Wallace, and modified Priestley-Taylor evapotranspiration models for wildland vegetation in semiarid rangeland. Water Resour. Res. 29, 1379–1392. doi: 10.1029/93WR00333

[B38] Sun Z. Wang Q. Ouyang Z. Watanabe M. Matsushita B. Fukushima T. (2007). Evaluation of MOD16 algorithm using MODIS and ground observational data in winter wheat field in North China Plain. Hydrol. Processes 21, 1196–1206. doi: 10.1002/hyp.6679

[B39] Todorovic M. (1999). Single-layer evapotranspiration model with variable canopy resistance. J. Irrigation Drain. Eng. 125, 235–245. doi: 10.1061/(ASCE)0733-9437(1999)125:5(235

[B40] Unland H. E. Houser P. R. Shuttleworth W. J. Yang Z. L. (1996). Surface flux measurement and modeling at a semi-arid Sonoran Desert site. Agric. For. Meteorol. 82, 119–153. doi: 10.1016/0168-1923(96)02330-1

[B41] Wang J. Wang J. Liu J. Jiang Y. Wang G. (2016). Calibration and evaluation of R-K evapotranspiration model for winter wheat in North China Plain. Trans. Chin. Soc. Agric. Eng. 32, 99–105. doi: 10.11975/j.issn.1002-6819.2016.09.014

[B42] Wei Z. Liu Y. Xu D. Cai J. Zhang B. (2013). Application and comparison of winter wheat canopy resistance estimation models based on the scaling-up of leaf stomatal conductance. Chin. Sci. Bull. 58, 2909–2916. doi: 10.1007/s11434-013-5858-3

[B43] Wu Z. Cui N. Zhao L. Han L. Hu X. Cai H.. (2022). Estimation of maize evapotranspiration in semi-humid regions of northern China using Penman-Monteith model and segmentally optimized Jarvis model. J. Hydrol. 607, 55–66. doi: 10.1016/j.jhydrol.2022.127483

[B44] Xing L. Cui N. Liu C. Guo L. Zhao L. Wu Z.. (2024). Estimating daily kiwifruit evapotranspiration under regulated deficit irrigation strategy using optimized surface resistance based model. Agric. Water Manage. 295, 108745. doi: 10.1016/j.agwat.2024.108745

[B45] Xu J. Liu X. Yang S. Qi Z. Wang Y. (2017). Modeling rice evapotranspiration under water-saving irrigation by calibrating canopy resistance model parameters in the Penman-Monteith equation. Agric. Water Manage. 182, 55–66. doi: 10.1016/j.agwat.2016.12.010

[B46] Xu J. Wu B. Ryu D. Yan N. Zhu W. Ma Z. (2021). Quantifying the contribution of biophysical and environmental factors in uncertainty of modeling canopy conductance. J. Hydrol. 592. doi: 10.1016/j.jhydrol.2020.125612

[B47] Xu J. Wu B. Yan N. Tan S. (2018). Regional daily ET estimates based on the gap-filling method of surface conductance. Remote Sens. 10. doi: 10.3390/rs10040554

[B48] Yan H. Yu J. Zhang C. Wang G. Huang S. Ma J. (2020). Comparison of two canopy resistance models to estimate evapotranspiration for tea and wheat in southeast China. Agric. Water Manage 245. doi: 10.1016/j.agwat.2020.106581

[B49] Yan H. Zhou Y. Zhang J. Wang G. Zhang C. Yu J.. (2022). Parametrization of canopy resistance and simulation of latent heat fluxes for typical crops in southern Jiangsu Province. Trans. Chin. Soc. Agric. Eng. 38, 101–107. doi: 10.11975/j.issn.1002-6819.2022.09.011

[B50] Zhang B. Kang S. Li F. Zhang L. (2008). Comparison of three evapotranspiration models to Bowen ratio-energy balance method for a vineyard in an arid desert region of northwest China. Agric. For. Meteorol. 148, 1629–1640. doi: 10.1016/j.agrformet.2008.05.016

[B51] Zhang K. Kimball J. S. Running S. W. (2016). A review of remote sensing based actual evapotranspiration estimation. Wiley Interdiscip. Rev.: Water 3, 834–853. doi: 10.1002/wat2.2016.3.issue-6

[B52] Zhang C. Li L. Yan H. Ou M. Akhlaq M. Zhang W.. (2022a). Calibration and validation of three evapotranspiration models in a tea field in the humid region of south-east China. Irrigation Drain. 71, 1254–1267. doi: 10.1002/ird.2728

[B53] Zhang D. Mak-Mensah E. Zhou X. Wang Q. Obour P. B. (2022b). Impact of plastic film with wheat straw mulching on maize water use efficiency, evapotranspiration, and grain yield in northern China: a meta-analysis. J. Soil Sci. Plant Nutr. 23, 867–880. doi: 10.1007/s42729-022-01089-z

[B54] Zhao H. Shen S. Hua R. Tao S. Zhang X. (2015). Simulation of canopy resistance for paddy rice based on Penman-Monteith model. Chin. J. Agrometeorol. 36, 17–23. doi: 10.3969/j.issn.1000-6362.2015.01.003

[B55] Zheng S. Wang T. Wei X. (2022). Estimating grapevine transpiration in greenhouse with three different methods in a Penman–Monteith model in Northeast China. Irrigation Sci. 40, 13–27. doi: 10.1007/s00271-021-00753-z

[B56] Zhou S. Yu B. Huang Y. Wang G. (2015). The complementary relationship and generation of the Budyko functions. Geophys. Res. Lett. 42, 1781–1790. doi: 10.1002/2015GL063511

